# Sequence Analysis of Pvama-1 among *Plasmodium Vivax* Isolates in Sistan-Baluchistan

**DOI:** 10.4314/ejhs.v30i4.6

**Published:** 2020-07-01

**Authors:** Hadi Mirahmadi, Tahere Safari, Malihe Metanat, Seyed Mehdi Tabatabaei, Ahmad Mehravaran, Saber Raeghi

**Affiliations:** 1 ^3^Infectious Diseases and Tropical Medicine Research Center, Resistant Tuberculosis Institute, Zahedan University of Medical Sciences, Zahedan, Iran; 2 Health Promotion Research Center, Zahedan University of Medical Sciences, Zahedan, IR Iran; 3 Department of Parasitology and Mycology, Faculty of Medicine, Zahedan University of Medical Sciences, Zahedan, Iran; 4 Department of Laboratory Sciences, Maragheh University of Medical Sciences, Maragheh, Iran

**Keywords:** Plasmodium vivax, PvAMA-1, Iran, Genetic variation, Sistan

## Abstract

**Background:**

Apical Membrane antigen 1 (AMA-1) is an important membrane protein that presents in all Plasmodium species and participates in critical phases in the attraction of cells. In human, it is one of the most immunodominant antigens with a protective immune response simulation role Apical Membrane antigen 1 (AMA-1) is an important membrane protein which presents in all Plasmodium species and is located on the surface of merozoite and sporozoites that participates in critical phases in attraction of human red blood cells by merozoites and hepatocytes by sporozoites, so in human, it is one of the most immunodominant antigens with a protective immune response simulation role. Since extra information is necessary to lighten of AMA-1 scope, we equaled genetic variation in P.vivax AMA-1 from 40 Iranian isolates with those reported from the other malarious countries.

**Methods:**

Blood samples were collected from 40 patients' positive of P.vivax, and genomic DNA was extracted from the blood. The nucleotide sequence for 446 amino acid (AA) residues (42–488 of PvAMA-1) of AMA-1 gene was amplified via PCR and then sequenced.

**Result:**

A total of 24 different haplotypes were recognized between samples. No new haplotype was determined in this research that was reported previously in other regions of Iran and the world. We detected 37-point mutations at the nucleotide level in their sequences and showed 43 amino acid variations, at 37 positions in which 6 sites demonstrate trimorphic polymorphism, and the others were dimorphic.

**Conclusion:**

Sequence analysis of the major haplotype showed 95% similarity with P.vivax Sal-1 AMA-1 gene and high level of allelic diversity at the domain I of PvAMA-1 among *P. vivax* isolates of Iran. Because PvAMA-1 is noticeable as vaccine candidate antigen, these documents provide valuable information for the development of malaria vaccine.

## Introduction

Malaria is a major public health problem in Middle East and tropical countries. Iran is located in the Middle East Region with low endemicity for malaria that occurs in south and southeast provinces in Iran (Hormozgan andSistan-Baluchistan). Both indigenous and imported malaria were described in these regions (1,2). Commonly, infection with *P. vivax* occurs in these areas in 90% of cases (3,4). More than 40% of people around the world are in danger of exposure to malaria. Approximately 250 million clinical cases and 6000 deaths due to malaria are reported every year worldwide. Though most of these deaths are related to *P. falciparum*, *P. vivax* has the main load of the disease because of its complications like anemia, respiratory distress, coma, and even death.(5).

In spite of combined therapies against malaria, It is important that studies be done to make the right vaccine. (6).

*Plasmodium* species pass their life cycle by infecting host RBC and cause disease. This parasite causes contamination of the host cells by the production of protein antigens (7), and it seems Apical Membrane Antigen 1 (AMA-1) has the main role in this process (8,9). AMA-1 is a type 1 membrane integral protein, the extracellular portion of it has three major domains with eight intramolecular di-sulfide-bonds (10–12). Although the biological activity of AMA-1 is unknown, several studies have confirmed its role in binding to the host's red blood cells (13). Antibodies against AMA-1 can prevent infection of host RBC by *P. falciparum* species. Due to the commonality of AMA-1 between *P. falciparum* and *P. vivax*, we can consider this antigen as the most important factor for developing a good vaccine against malaria species (14,15).

One of the serious barriers in the way of developing vaccine against malaria is the high genetic diversity (16). In a study in Mali, provided vaccine was effective against one species of *P.falciparum* only (17), and in another study, the bivalent vaccine provided no protection (18). At the moment, multivalent vaccines also have a high risk of immune cross-reactions (19,20). All these reasons represent that the genetic diversity of AMA-1 allele and indicate the *P.vivax* has more diversity than *P. falciparum*. The studies have shown that due to the biological differences between *P. falciparum* and *P. vivax* species, we cannot generalize the results of a species to another species (21,22).

As a result, sequencing and understanding of the AMA-1 gene sequence in P.vivax will be necessary independently in endemic areas that are aimed at eradicating both species in order to develop the appropriate vaccine (23). In the recent research, the PvAMA-1 was sequenced and then compared with similar genes and tried to compare the mutations related to each AMA-1 domain of I, II and III, with each other carefully.

## Methods And Materials

### Study areas and blood samples collection

Giemsa stained thick and thin blood smears were collected from 40 referred confirmed patients positive of *P. vivax* by light microscopic analysis from healthcare centers in the study regions (Sistan-Bluchestan Province) to Zahedan Health Centers. After earning consent, the samples transformed to parasitology lab until DNA extraction.

### DNA extraction and PCR amplification

**Figure 1:**
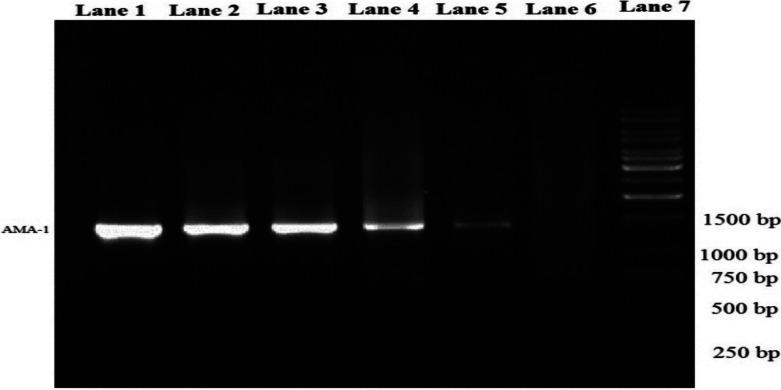
PCR electrophoresis on gel agarose: positive control (1), positive samples (2-5), negative control (6), size marker 1000 bp (7)

DNA was extracted by DynaBio Blood/Tissue Genomic DNA extraction kit (TAKAPUZIST®) according to manual. The quantity and quality of extracted product was tested via spectrophotometer at 260 and 280 nm and electrophoresis on 1% agarose gel. The PvAMA-1 gene, including domain I, domain II and domain III, expressing amino acids (AA) from 42–488 was aimed for amplification. The primers were selected based on the sequence of *P. vivax* Sal-1 PvAMA-1gene (ACCESSION NO.: XM_001615397). The amplification for PCR was performed in a 200 µl PCR tube containing: 200 ng (2 µl) extracted DNA as template, 25 pmol(1 µl) of each primer, 7.5 µl of PCR master mix (Roche) and ddH2O up to 15 µl. The targeted gene was amplified for 30 cycles as follows: initial denaturation at 95°C for 5 min followed by 30 cycles with denaturation at 94 °C for for 20s, annealing at 58◦C for 30s and extension at 72 °C for 2 min and final extension at 72 °C for 5 min), PCR product was 1330 bp (Figure 1) which contained domain I, domain II and domain III of the PvAMA-1 gene with PvAMA F (5′ CCATGGGGCCTACCGTTGAGAGAA-3′) and PvAMA R as (5′CTCGAGTCATAGTAGCATCTGCTTGTT-3′) primers (24). The product was analyzed on 1% agarose gel against size marker (Fermentase®).

### Sequence analysis

Received sequencing results along with the chromatogram from Bioneer (Korea) check and were ordered by software Geneious. Nucleotide sequences were analyzed by Chromas 2.2 software and aligned by ClustalW program. The 40 Iranian sequences were compared with formerly consigned *P. vivax* AMA-1 in the GenBank. The nucleotide diversity and number of polymorphic sites (S), as well as the number of haplotypes (H) were obtained by DNASP software. Nucleotide variation and statistical analysis were investigated by MEGA software.

AMA-1 gene of *P. vivax* Sal1 strain (Accession No. XM_001615397), as complete sequence, was used to find nucleotide and amino acid position numbers. The *Plasmodium falciparum* (Accession No.X86099) was used as an outgroup species to analyze impartiality tests. Maximum Likelihood (ML) algorithm using MEGA7 software. All characters were run unordered and equally weighted. Alignment gaps were treated as missing data. Bootstrap analysis was conducted using 1000 replicates.

### Ethical considerations

The experimental procedure was approved by the Committee of the Ethics on Zahedan University of Medical Sciences and performed in Infectious Diseases and Tropical Medicine Research Center.

## Results

**Figure 2:**
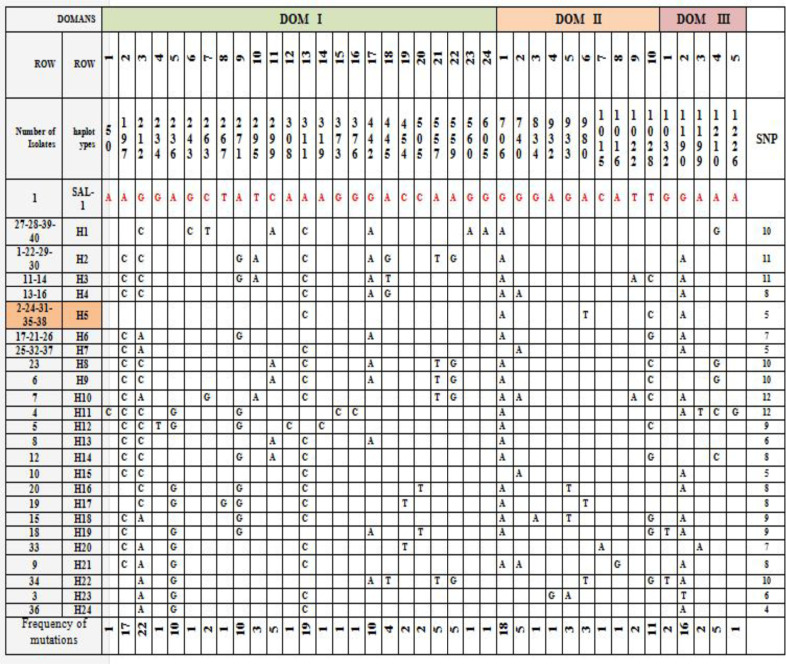
Single nucleotide polymorphisms (SNP) in Iranian isolates compared to Sal-1 isolate

A total of 40 isolate sequences of the AMA-1 gene was identified from patients confirmed by blood smear. The 1330 bp in length, belonging to domains I, II and III (respectively to 1–615, 616–1029 and 1030–1338 bp region of AMA-1 gene of Sal1 accession number: XM_001615397) was amplified by molecular methods (PCR) and sequenced among the Iranian Southeastern P.vivax isolates. There clearly was no recognized size polymorphism in PCR and all isolates exhibited in gel electrophoresis 1400 bp fragments approximately. Totally, 24 various haplotypes distinguished among 40 sequences (Figure 1). No new haplotypes were determined to cooperation with others haplotypes that were described previously in different loci of the world. Haplotypes 1, 2 with 4 copies and haplotype 5 with 5 copies demonstrated the most frequency, although the other 21 haplotypes from one to three copies were varied (Figure 2).

Totally, we observed 24, 8 and 5-point mutations at the nucleotide level in the analyzed sequences in Dom I, II and III respectively. In between, 1336 bp analyzed sequences the 37 variable (polymorphic) nucleotide sites were demonstrated that 16 sites of them are singleton and 21 sites were multiplex

**Figure 3:**
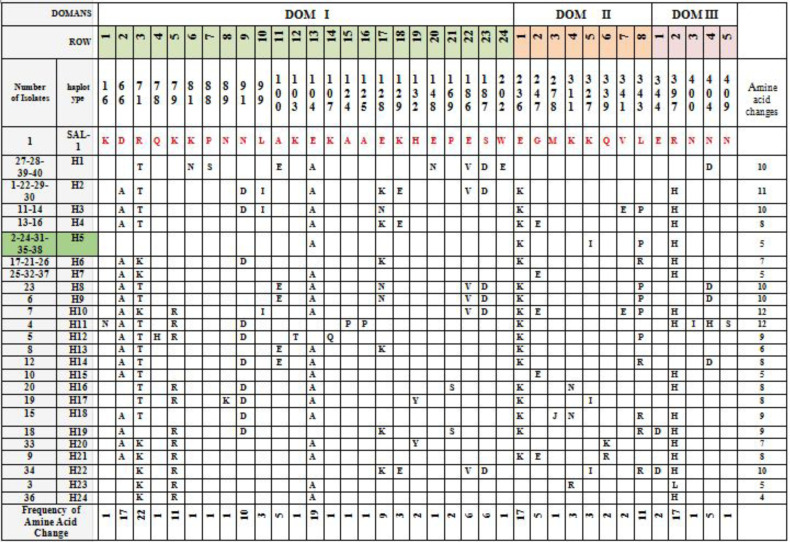
The amino acid changes compared to Sal-1 isolate referred to PvAMA-1 gene

**Figure 4:**
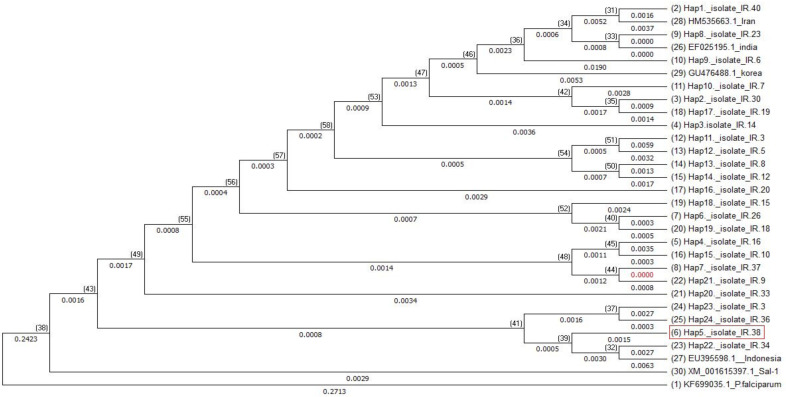
Neighbor-Joining tree of the AMA-1 haplotypes using of MEGA 7. Bootstrap values based on to 1000 replications. The haplotypes of this research (H1–H24) and a Strain of *P. vivax* Sal1 (Accession No. XM_001615397), and four isolates from several countries were used in phylogenetic construction, containing S3 EF025195: India, SKO814: Korea, EU395598: Indonesia. The sequence of AMA-1 in *P. falciparum* (Accession No.X86099) used as an outgroup

Among 37 variable nucleotides positions, six sites were trimorphic (3, 2 and 1 sites on Dom I, II and III respectively), the others of variable nucleotides positions were dimorphic. Nucleotide diversity and haplotypes of the sequenced PvAMA-1 are demonstrated in Figure 2. Sequences analyses showed 43 amino acid changes at 37 positions in which 6 sites (2, 3 and 1 sites on Dom I, II and III respectively) demonstrated trimorphic polymorphism, and the others were dimorphic (Figure 3). Also, phylogenic tree conducted by neighbor-joining (NJ) using MEGA 6.0 based on AMA-1 haplotypes is shown in Figure 4. The haplotypes of this research (H1–H24) and a Strain of *P. vivax* Sal1 (Accession No. XM_001615397), and four isolates from several countries were used in phylogenetic construction, containing S3 EF025195: India, SKO814: Korea, EU395598: Indonesia. The sequence of AMA-1 in *P. falciparum* (Accession No.X86099) used as an outgroup.

## Discussion

Infectious and zoonotic diseases have a great importance in the Middle East and Africa (25,26). Although the *Plasmodium vivax* is mostly benign, due to its geographical extent, it causes significant socio-economic burdens in endemic areas. *P. vivax* is chronic malaria and causing frequent relapse in people. In addition, it affects the working capacity of people in the community. The reason for the limitation in the study of parasitic diseases, especially *P. vivax*, is related to the lack of proper culture and obtaining appropriate and sufficient antigens, severe symptoms of the disease, including anemia..

This species is responsible for 80 million malaria infections every year in the world. Due to its different variants, the detection of parasite genome for vaccine, diagnostic kits, drug therapy and subsequently control of the disease are essential..

AMA-1 is a member of a group of molecules that have been completely preserved in Plasmodium species (30), and it is expressed in the large scale on the surface of the merozoite of all species of Plasmodium and in the micronemes of them. This antigen plays a unique role, during the vital stages of the invasion of sporozoite cells into the liver and merozoite into red blood cells (31). The number of haplotypes is limited and exposes a limited diversity in each geographic region. In this study, 40 sequenced isolates from endemic malaria areas in a one-year period were analyzed and compared with deposited *P. vivax* AMA-1 in the GenBank from malaria countries in Asia

In the target gene, the region of this study is 1338 bp, and the translated protein sequence contains 446 amino acids, which have 16 cysteine sites. Using Blast and ClustalW software, dominant haplotype compared to previously deposited *P. vivax* AMA-1 in the GenBank database, which showed the highest homology with Indonesian isolates with a similarity of 99%. The analysis of nucleotides in comparison to this isolate led to a change in the 9 amino acids. In the dominant haplotype changes on amino acid level were 11 cases which it was the least homology with Korea isolates. At the level of amino acids, most of these differences are observed in the DomI. In Iranian isolates, the Dom II with 3 amino acids was completely different from the Sal-1 isolate and these changes lead to the diversity of parasitic populations in different geographic regions. The dominant Iranian haplotype in the Dom II on 327 amino acid position (980 nucleotide position) was different with all strains and could be considered as an indicator in the detection of Iranian isolates. In our study, Dom II was high difference with the previous reports of malaria. The Dom I and II are able to provide protection to the person therefore these markers are useful for assessing the presence of antimalarial antibodies in people living in endemic areas. This area can be a good candidate for immunological studies as well as a component in the provision of a proactive vaccine. The dominant Iranian haplotype at the level of amino acids, the least of these differences were in the Dom III with 1 difference compared to the Sal-1 isolate. This domain is responsible for cross-reactivity between species.

According to previous studies, the building of PvAMA-1 by 16 cysteine sites and the creation of disulfide bonds is stabilized. Cysteines are essential to stabilization of produced protein formation and specific antibodies against this protein.

A similar study was conducted in Brazil and studied the variation of PvAMA-1 in northern Brazil. The number of tested samples in this study was 20, with more than 93% identity. In this study, 40 samples were examined and 90% of the homology was observed (32).

The only study in Iran was conducted by Motevalli Haghi that studied the variation of PvAMA-1 in southern Iran. The number of samples tested in this study was 6, with more than 94% identity. In this study, samples were examined and 90% of the homology was observed (24).

The other study was conducted in Honduras with 85 cases and identified the genetic variation of PvAMA-1 genes which were a length of 466 nucleotides and encoded 151-154 amino acids (33). Also, a study from Sri Lanka had shown the genetic diversity in Dom II of PvAMA-1 and confirm 11 variations in the amino acid position as well as 21 haplotypes with 9 distinct haplotypes in the PvAMA-II gene (34).

In a study from South Korea, 30 haplotypes of *P. vivax* were identified, while the DOM II was most protected and the Dom I and III had the highest mutations. In a study in South Korea, 66 samples of blood from infected with P.vivax, 30 haplotypes were identified from seven different cluster isolates while the DOM II was most protected and the Dom I and III had the highest mutations (35).

In conclusion, since the AMA-1 recombinant protein is a hopeful candidate for development of vaccine, applying it into polyvalent vaccine as a particular member might generate immune reactions more effective against parasites. Thus, this research along with previous research on the PvAMA-1 locus on the basis of the local recognition of AMA-1 sequences could be so effective in guarding against

P. vivax in malarious regions of Iran. In conclusion, we analyzed the genetic diversity of PvAMA-1 between Iranian isolates. Our data indicate moderately high level of nucleotides and haplotype diversity at the PvAMA-1 between *P. vivax* isolates of Iran. Since PvAMA-1 is considered as vaccine candidate antigen, these data are valuable for the development of a PvAMA-1 based malaria vaccine.
